# Long-term results after surgical resection of intrahepatic cholangiocarcinoma before and since the BILCAP era

**DOI:** 10.3389/fsurg.2026.1853219

**Published:** 2026-06-10

**Authors:** Fabian Bartsch, Constantin Scholz, Lisa-Katharina Gröger, Lara Bachmann, Janine Baumgart, Ann-Kathrin Lederer, Jens Mittler, Maria Hoppe-Lotichius, Beate K. Straub, Friedrich Foerster, Arndt Weinmann, Evangelos Tagkalos, Hauke Lang

**Affiliations:** 1 Department of General, Visceral and Transplant Surgery, University Medical Center of The Johannes Gutenberg-University Mainz, Mainz, Germany; 2 Department of General and Visceral Surgery, Hospital ‘Barmherzige Brüder’ Trier, Trier, Germany; 3 Department of Pathology, University Medical Center of The Johannes Gutenberg-University Mainz, Mainz, Germany; 4 1st Department of Internal Medicine, Gastroenterology and Hepatology, University Medical Center of The Johannes Gutenberg-University Mainz, Mainz, Germany

**Keywords:** adjuvant therapy, BILCAP, capecitabine, cholangiocarcinoma, intrahepatic cholangiocarcinoma, liver surgery, overall survival, recurrence

## Abstract

**Background:**

Since the first data of the BILCAP trial was presented in 2017, capecitabine became standard adjuvant treatment for intrahepatic cholangiocarcinoma (ICC). For ICC, the efficacy of capecitabine on survival outcomes remains unresolved as the BILCAP trial included all entities of biliary tract cancer. The aim of this study was to evaluate the influence of adjuvant therapy with capecitabine on long-term outcome in a large German single-center cohort.

**Methods:**

All patients who underwent surgical resection of ICC between January 2008 and December 2023 were divided into a pre-BILCAP (2008–2017) and a post-BILCAP group (2018–2023). For further homogenization both groups underwent a propensity score matching (PSM) in a 1:1 fashion. Survival analysis was conducted using Kaplan Meier model.

**Results:**

In total 334 patients were included, 254 (76%) underwent resection while 80 (24%) were irresectable. After PSM two comparable groups of 75 patients were generated. Median overall survival was 20.5 months for the pre- and 29.1 months for the post-BILCAP group (*p* = 0.351). Time to recurrence (TTR, median 10.8 versus 21.3 months, *p* = 0.019) and recurrence-free survival (RFS, median 8.6 and 10.7 months, *p* = 0.029) were significantly better for the post-BILCAP group.

**Conclusion:**

Although there was a trend to longer survival, we could not demonstrate a significant effect of adjuvant therapy with capecitabine on an intention to treat basis after resection of intrahepatic cholangiocarcinoma. Nevertheless, a significant influence on time to recurrence and recurrence-free survival could be demonstrated. Further observation is necessary to detect the potential benefit of decreased RFS/TTR in the future.

## Introduction

Intrahepatic cholangiocarcinoma (ICC) is a rare and in most cases lethal malignancy. It is often diagnosed in an advanced stage and complete resection is the only curative therapy. Tumor recurrence is common with varying rates within the literature leading to poor 5-year overall survival rates of 20%–40% (1–5).

Over the last decades liver surgery evolved rapidly, enabling the implementation of extended resection as standard procedure, even for intrahepatic cholangiocarcinoma (6–9). But overall survival did not improve essentially. French data from 1999 reported about a 3-year survival of 22% (10), while Japanese data from 2001 achieved a 5-year survival of 28% (11). Offering patients with advanced tumors a potentially curative resection led to a stagnation of long-term survival rates. While surgery cannot influence tumor biology, multidisciplinary approaches are urgently needed.

Before 2010 data regarding neoadjuvant, adjuvant and palliative chemotherapy for ICC were generally scarce. In 2017 first results of the BILCAP study were presented by Primrose et al. at the annual meeting of the ASCO (American Society of Clinical Oncology) (12) and capecitabine was implemented as the standard of care adjuvant therapy after curative intended resection. The whole study was published in 2019 and often criticized or questioned because significancy was only reached in the per protocol analysis while the intention to treat analysis did not (13). The long-term outcome was published in 2022 endorsing the earlier results (14).

The aim of this study was to compare long-term overall survival (OS), time to recurrence (TTR) and recurrence-free survival (RFS) of patients who underwent resection before and since the BILCAP era. Potential influence of adjuvant application of capecitabine should be analyzed in a large German intention to treat single-center cohort.

## Methods

This retrospective single-center study analyzed a cohort of patients who underwent exploration for intrahepatic cholangiocarcinoma within a period of 16 years (2008 to 2023). Exclusion criteria for patients were as follows: age under 18 years; suffering from other primary liver tumors like hepatocellular carcinoma, other malignancies like perihilar cholangiocarcinoma or gallbladder carcinoma and all secondary metastases were excluded.

The preoperative workup involved computed tomography (CT) or magnetic resonance imaging (MRI) performed in our center. Even external imaging was accepted if quality criteria were met. Regularly measured tumor markers included carbohydrate antigen 19-9 (CA19-9) and carcinoembryonic antigen (CEA). Liver function was determined through serum values of bilirubin, quick/INR and protein levels (e.g., albumin). All patients were preoperatively discussed in an interdisciplinary conference composed of experienced liver surgeons, radiologists and oncologists.

Candidates for resection had exclusion of distant metastasis and showed no signs of portal hypertension in preoperative imaging, had an apparently well liver function considering laboratory values, and fulfilled the technical aspect of resectability. Resections were performed by an experienced and consistent team of hepato-pancreatico-biliary surgeons. Patients who were unresectable at surgical exploration were excluded from all analysis. Follow-up after resection was performed every three months, at least for two years after resection. A CT scan or MRI was conducted every six months in regular three months alternation with ultrasound examinations. If the patients were not able or willing to undergo follow-up at our center, information was retrieved from treating physicians. Follow-up completeness was >90%. Some patients were lost to follow-up by changing their physician and/or moving away. If patients were lost to follow-up, we censored survival or recurrence on the date of last information obtained.

While the first results of the BILCAP study were presented at the ASCO 2017 we assumed that some time was necessary for complete application of capecitabine as standard adjuvant treatment after resection of ICC. All patients got an explicit recommendation to undergo adjuvant treatment with capecitabine. Therefore, we defined the pre-BILCAP group from the year 2008 until the end of 2017, while the post-BILCAP group started from 2018 until the end of 2023. Some patients underwent neoadjuvant therapy before resection. These patients also got the recommendation to undergo adjuvant therapy with capecitabine.

We decided to perform an analysis depending on time-periods instead of a treatment-based comparison. This is because the cohort/database has a surgical focus and data regarding adjuvant administration of capecitabine is not complete. Patients who underwent surgery in our department came from all over Germany, some even internationally. Due to the low incidence of intrahepatic cholangiocarcinoma, number of patients who underwent adjuvant therapy and complete follow-up at our center is low. Follow-up focused on tumor recurrence and survival, and we could not acquire full medical reports of all patients. Due to the retrospective design of this study, we cannot offer data regarding treatment exposure with capecitabine, dose, completion rates or any suffered toxicity. Therefore, this study relies on the intention to treat and should be interpreted accordingly.

Data of patients undergoing resection was further analyzed regarding preoperative findings, performed resection and postoperative course, pathological findings, tumor recurrence, as well as recurrence-free and overall survival. Morbidity was assessed according to the Clavien-Dindo classification (15, 16). For TNM staging we used the 8th edition of the UICC classification [Union for International Cancer Control (17)].

According to Punt and colleagues (18), recurrence-free survival (RFS) was defined as an event occurring in case of tumor recurrence or at time of death; for time to recurrence (TTR) only tumor recurrence was defined as event, and all other patients were censored even in case of death.

All patients included in this study signed informed consent that data and follow-up will be collected anonymously and potentially used for scientific analysis. Abiding by the regulations of the federal state law (state hospital law §36 & §37) and according to the independent ethics committee of the state medical association of Rhineland-Palatinate, no ethical approval was necessary, and no approval number was assigned for this study. The research was conducted according to the Declaration of Helsinki.

Statistical analyses were performed using SPSS 29 (IBM Corp. Released 2023. IBM SPSS Statistics for Windows, Version 29.0. Armonk, NY: IBM Corp). For categorical data the Chi^2^ test was used in cross tabulation or a t-test to compare means of two groups. Statistical tests were given with respective Test statistics and degree of freedom. Analyses of time to recurrence (TTR), recurrence-free (RFS) and overall survival (OS) were conducted using the Kaplan Meier model and for comparison of influencing factors log rank test was utilized. Significance was considered with a *p*-value of <0.05. *P*-values were given with 95%-Confidence intervals.

To reduce the bias of retrospective analysis and to adjust the pre-BILCAP and post-BILCAP groups, propensity score matching (PSM) was applied in a 1:1 fashion. Patients with incidental intraoperative finding of an M1 situation were excluded from the cohort for PSM. Matching was performed manually with a caliper width of ±0.02 and without replacement. All pre-selected, relevant confounders are included in the logistic regression model simultaneously using the ‘enter’ model. Parameters used for logistic regression within the context of PSM were expected to be related to the outcome. We decided to include T-stage, N-stage, Grading, R-status, and major resection as confounding variables. Tumor size and multifocality were not considered due to their consideration in the T-stage. Balance diagnostics are provided as standard mean differences within the results.

## Results

**Figure 1:**
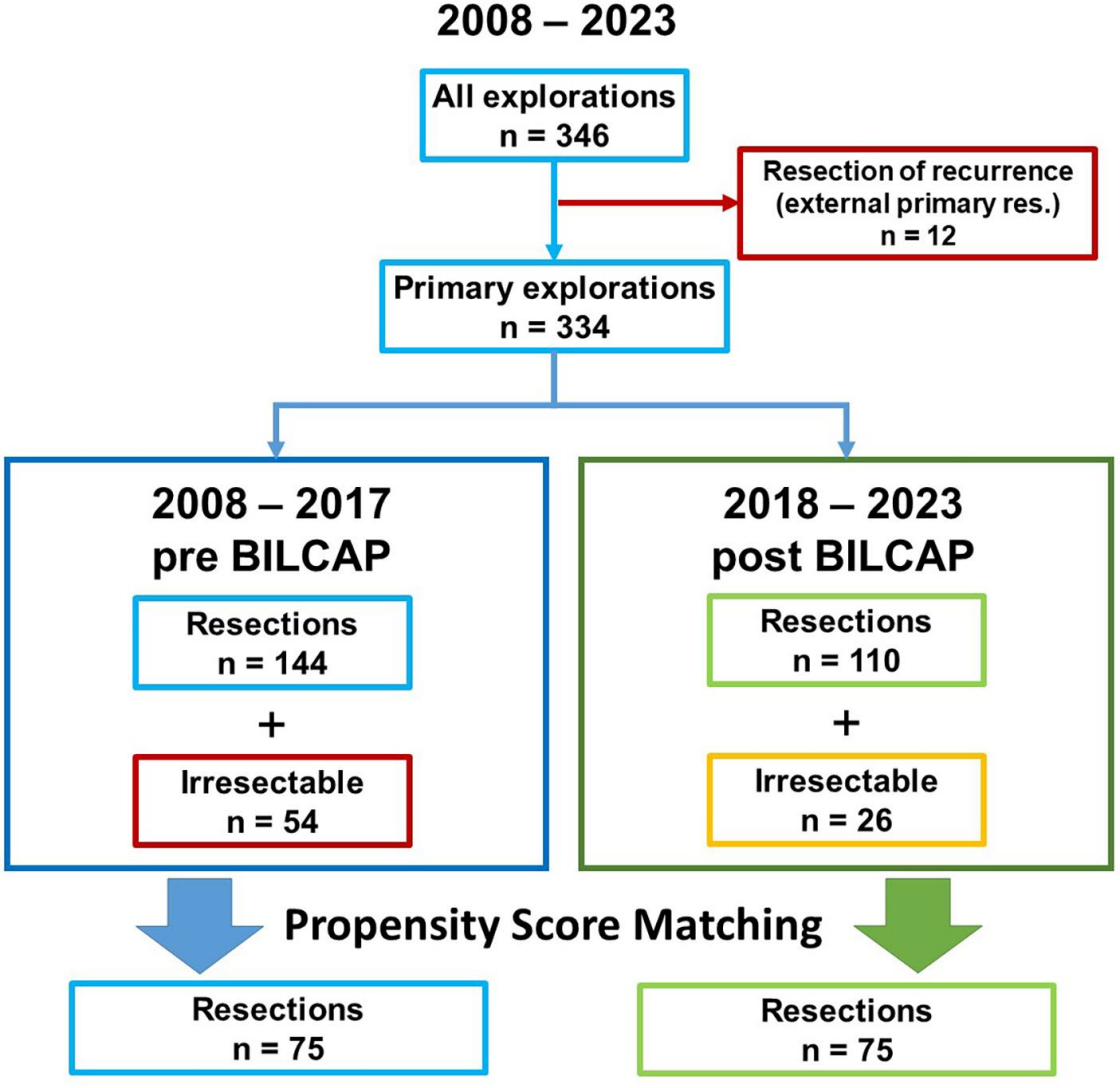
Flow chart of the study cohort and subgroups before and after propensity score matching. Irresectable patients were excluded from all analyses.

Within the study period 346 patients underwent surgical exploration for intended liver resection for ICC of whom 334 were included in this study; 254 (76%) underwent resection and 80 (24%) were irresectable. Figure 1 demonstrates the further subdivision into the two essential resection groups within the pre-BILCAP and post-BILCAP era.

### Surgical procedures, histological outcome and propensity score matching

The main demographics, physical performance status, types of performed resections and histological results are shown in Table 1. For focality (*p* = 0.002), tumor size (*p* = 0.032), T-status (*p* = 0.012) and R-status (*p* = 0.004) significant differences were apparent. Twentyfour patients underwent neoadjuvant therapy [chemotherapy (*n* = 22), TACE (*n* = 1), ablation (*n* = 1)].

**Table 1:** Patients characteristics.

	All patients	pre-BILCAP	post-BILCAP	*p*-value
		2008–2017	2018–2023	
	*n* = 254	*n* = 144	*n* = 110	
Demografic and clinical baseline data				
Age [median (range)]	65.2 (28.8–84.4)	64.1 (32.3–84.4)	68.2 (28.8–84)	0.056
Gender (female/male)	127/127	72/72	55/55	1
BMI [median (range)]	26.1 (15.1–48.8)	26.1 (15.1–43.1)	26 (17–48.8)	0.746
ASA				0.632
I	3	1	2	
II	112	62	50	
III	132	78	54	
IV	7	3	4	
Procedures				
Major resection	198	106	88	0.235
ALPPS	8	7	1	
Extended right hepatectomy	37	25	12	
Extended left hepatectomy	39	20	19	
Right hepatectomy	33	24	9	
Left hepatectomy	45	18	27	
Mesohepatectomy	16	7	9	
Resection of three liver segments	20	9	11	
Minor resection	56	38	22	
Bisegmentectomy	39	24	15	
Monosegmentectomy	15	8	7	
Atypical resection	2	2	-	
Histology				
Solitary/multifocal tumor	191/63	98/46	93/17	0.002
Tumor size in cm * [median (range)]	6.5 (0.4–20)	7 (0.4–20)	6.5 (0.5–17)	0.032
Lymph nodes harvested [median (range)]	5 (0–31)	4 (0–31)	5 (0–21)	0.513
T-status				
T1a	46	20	26	0.012
T1b	68	31	37	
T2	91	58	33	
T3	18	13	5	
T4	31	22	9	
N-status				0.407
N0	157	85	72	
N1	72	42	30	
Nx	25	17	8	
M-status				0.769
M0	239	136	103	
M1	15	8	7	
Grading				0.064
G1	4	2	2	
G2	164	85	79	
G3	60	44	16	
G4	2	1	1	
No grading after neoadj. Therapy (*N*AT)	24	12	12	
NAT: chemo/TACE/RFA	22/1/1	10 ^a^/1/1	12 ^b^/0/0	
R-status				0.004
R0	202	125	77	
R1	52	19	33	

* Size of the largest nodule.

a applied regimens: Gemcitabine/Cisplatin (*n* = 8), Gemcitabine (*n* = 2).

b applied regimens: Gemcitabine/Cisplatin (*n* = 7), Gemcitabine/Cisplatin/Durvalumab (*n* = 1), Gemcitabine/Cisplatin/Durvalumab/Tremelimumab (*n* = 1) FOLFIRINOX (*n* = 2), Cisplatin (*n* = 1).

Propensity score matching was applied for adjustment and better comparability. Patients with M1 status were excluded from PSM. These 15 patients presented with incidentally found solitary peritoneal noduli which were resected and found to be metastatic seeds in final histology. After PSM the pre- and post-BILCAP groups consisted of 75 patients, respectively. The distribution of parameters after PSM is presented in Table 2**,** including standard mean differences before and after PSM and *p*-values showing no significant differences anymore.

**Table 2:** Patients characteristics after propensity score matching.

	pre-BILCAP	post-BILCAP	SMD		*p*-value
	2008–2017	2018–2023	before	after	
	*n* = 75	*n* = 75	PSM^a^	PSM	
Demografic and clinical baseline data					
Age (mean/median)	66.1/66.7	67.9/69	−0.3	0.17	0.293
Gender (female/male)	34/41	33/42	−0.01	−0.03	0.870
BMI (mean/median)	26.8/ 26.1	26.8/26.4	0.02	0	0.993
ASA			0.04	−0.1	
I	1	1			0.521
II	24	30			
III	48	39			
IV	2	4			
Procedures					
Major resection	**58**	**59**	**0**.**14**	**−0**.**03**	**0**.**844**^b^
ALPPS	3	-			
Extended right hepatectomy	14	10			
Extended left hepatectomy	9	14			
Right hepatectomy	15	5			
Left hepatectomy	8	18			
Mesohepatectomy	4	6			
Resection of three liver segments	5	6			
Minor resection	**17**	**16**			
Bisegmentectomy	13	10			
Monosegmentectomy	3	6			
Atypical resection	1	-			
Histology					
Solitary/multifocal tumor	57/18	63/10	0.41	−0.26	0.110
Tumor size in cm * (mean/median)	7.7/7	6.9/6.5	0.24	−0.2	0.198
Lymph nodes harvested (mean/median)	5.4/3.5	4.5/5	−0.15	0.2	0.134
T-status					
T1a	14	11	**0**.**46**	**0**.**07**	**0**.**651**
T1b	22	28			
T2	29	23			
T3	5	5			
T4	5	8			
N-status					
N0	48	52	**0**.**2**	**−0**.**15**	**0**.**560**
N1	17	17			
Nx	10	6			
M-status					
M0	75	75	-	-	-
M1	-	-			
Grading					
G1	2	1	**0**.**11**	**0**.**01**	**0**.**170**
G2	45	53			
G3	21	10			
G4	0	1			
No grading after neoadj. Therapy (NAT)	7	10			
NAT: chemo/TACE/RFA	5^c^/1/1	10^d^/0/0			
R-status					
R0	61	61	**−0**.**36**	**0**	**1**
R1	14	14			

* Size of the largest nodule.

a Referring data is shown in Table 1.

b crosstabulation of major vs. minor resections.

c Gemcitabine/Cisplatin (*n* = 3), Gemcitabine (*n* = 2).

d Gemcitabine/Cisplatin (*n* = 7), Gemcitabine/Cisplatin/Durvalumab (*n* = 1), Gemcitabine/Cisplatin/Durvalumab/Tremelimumab (*n* = 1), FOLFIRINOX (*n* = 1).

SMD = standard mean difference; bold SMD and *p*-values are included parameters in PSM; NAT = neoadjuvant therapy.

### Morbidity and mortality

Distribution of morbidity and mortality in general as well as of the pre- and post-BILCAP groups before and after PSM are shown in Table 3. Out of all patients who underwent resection, 116 patients (45.7%) had no postoperative morbidity, 50 (19.7%) had minor (grade I + II) and 66 (25.9%) had major (grade IIIa – IVb) complications. Twenty-two patients (8.7%) deceased within 90 days postoperatively. Distribution of morbidity and mortality did not differ significantly between the groups before (*p* = 0.212) and after PSM (0.357).

**Table 3:** Morbidity and mortality

	all		no PSM		PSM	
			pre-BILCAP	post-BILCAP	pre-BILCAP	post-BILCAP
	n	%	*n* = 144	*n* = 110	*n* = 75	*n* = 75
Morbidity†						
no morbidity	116	45.7	73	43	40	27
Grade I	22	8.7	7	15	4	11
Grade II	28	11	16	12	8	9
Grade IIIa	44	17.3	26	18	7	12
Grade IIIb	9	3.5	3	6	2	3
Grade IVa	9	3.5	5	4	3	4
Grade IVb	4	1.6	2	2	1	1
Mortality						
Grade V	22	8.7	12	10	10	8
(30d/90d)	21/1		11/1	10/-	9/1	8/-

† Chi^2^ comparison before PSM *p* = 0.212 and after PSM *p* = 0.357.

### Recurrence

Within the whole cohort 152 of 254 patients (60.6%) sustained tumor recurrence; 103 (71.5% of 144 patients) in the pre-BILCAP and 49 (44.5% of 110 patients) in the post-BILCAP group. The ratio after PSM was 69.3% pre- and 44% post-BILCAP. Table 4 shows the distribution of isolated intra- or extrahepatic recurrence and the combination of both. Recurrence as well as the distribution of its localization were significantly different between the pre- and post-BILCAP groups before (both *p* < 0.001) and after PSM (*p* = 0.002 and 0.017, respectively).

**Table 4:** Tumor recurrence.

Resection group	*n* = 254	Recurrence n/%	Intrahepatic rec.	Extrahepatic rec.	Intra + extrahepatic rec.
		*n* = 152 59.8%	63	35	53
No PSM					
Pre-BILCAP	*n* = 144	*n* = 103 71.5%	43	25	35
Post.BILCAP	*n* = 110	*n* = 49 44.5%	20	10	18
*p*-value		<0.001^a^			<0.001^b^
PSM					
Pre-BILCAP	*n* = 75	*n* = 52 69.3%	20	14	18
Post-BILCAP	*n* = 75	*n* = 33 44%	14	7	12
*p*-value		0.002^a^			**0.017** ^b^

Significant *p*-values are bold.

a comparison of patients with and without recurrence, Chi^2^;.

b comparison of distribution of localization including no recurrence, Chi^2^.

### Survival analysis

The median and respective 1-, 3- and 5-year overall survival (OS) and time to recurrence (TTR) of the complete cohort as well as the pre- and post-BILCAP groups before and after PSM are shown in Table 5.

**Table 5:** Overall survival and time to recurrence.

n	All resections	pre-BILCAP	post-BILCAP	pre-BILCAP+PSM	post-BILCAP+PSM
	254	144	110	75	75
Overall survival					
Median (mo)	23.4	21.3	31.3	20.5	29.1
1-year (%)	72	71	73	71	73
3-year (%)	36	32	45	33	41
5-year (%)	22	19	31	17	24
*p*-value		0.075		0.291	
Time to recurrence					
Median (mo)	11.8	10.4	21.4	10.8	21.3
1-year (%)	49	43	58	44	57
3-year (%)	29	19	45	20	48
5-year (%)	25	17	42	20	42
*p*-value		**0.002**		**0.019**	

Significant *p*-values are bold.

**Figure 2:**
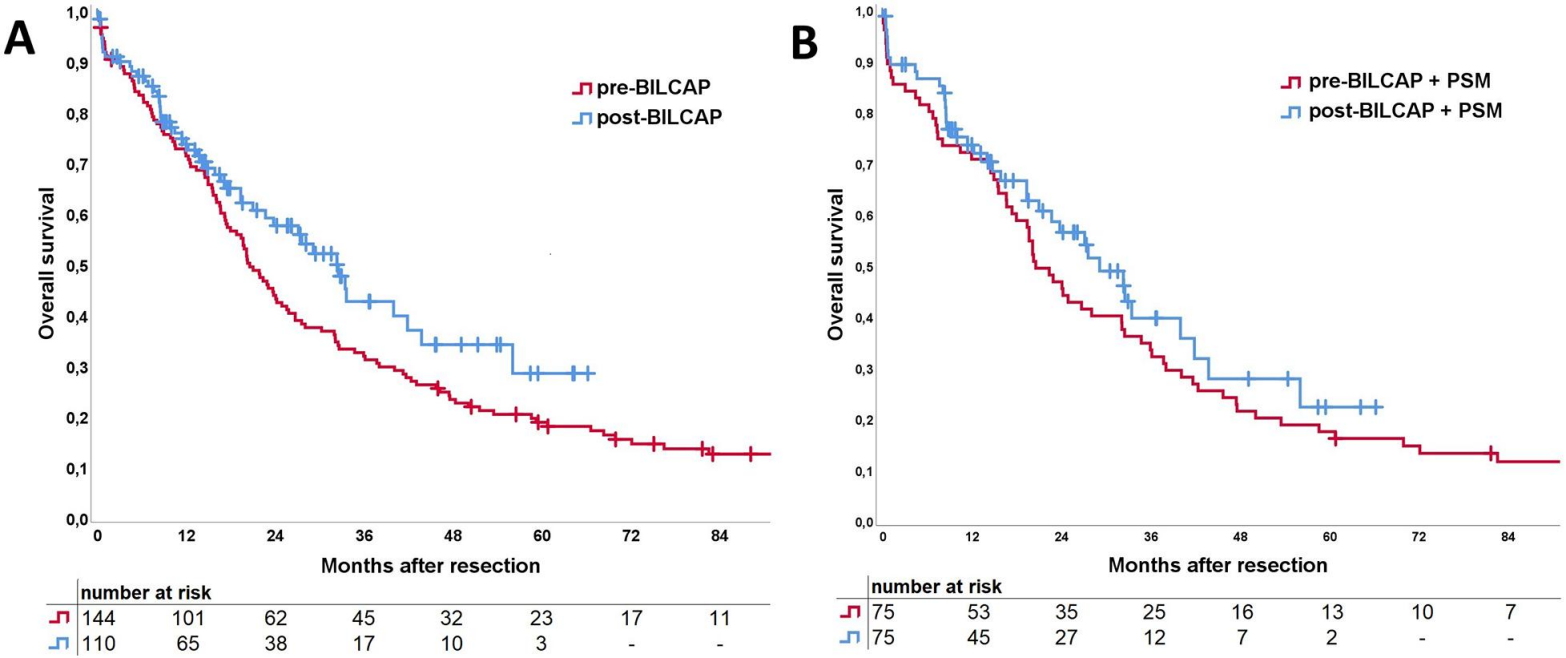
Kaplan Meyer curve comparing overall survival of **(A)** the initial pre- and post-BILCAP groups (*p* = 0.075) and **(B)** the pre- and post-BILCAP groups after propensity score matching (*p* = 0.291).

In comparison of OS the post-BILCAP group performed better with a median survival of 31.3 months (CI95% 25.4–39.0) vs. 21.3 months (CI95% 16.9–24.0) for pre-BILCAP, but without reaching statistical significance (*p* = 0.075, Figure 2A). After PSM the difference is less distinct and still not significant [pre-BILCAP median 20.5 months [CI95% 16.4–24.5] vs. post-BILCAP median 29.1 months [CI95% 20.3–37.8], *p* = 0.291, Figure 2B]. If perioperative deaths were excluded, the post-BILCAP group showed to be significantly better with a median OS of 35.8 months compared to 23.1 months in the pre-BILCAP group (*p* = 0.043). This effect did not reproduce for the PSM groups with excluded perioperative deaths (*p* = 0.351).

**Figure 3:**
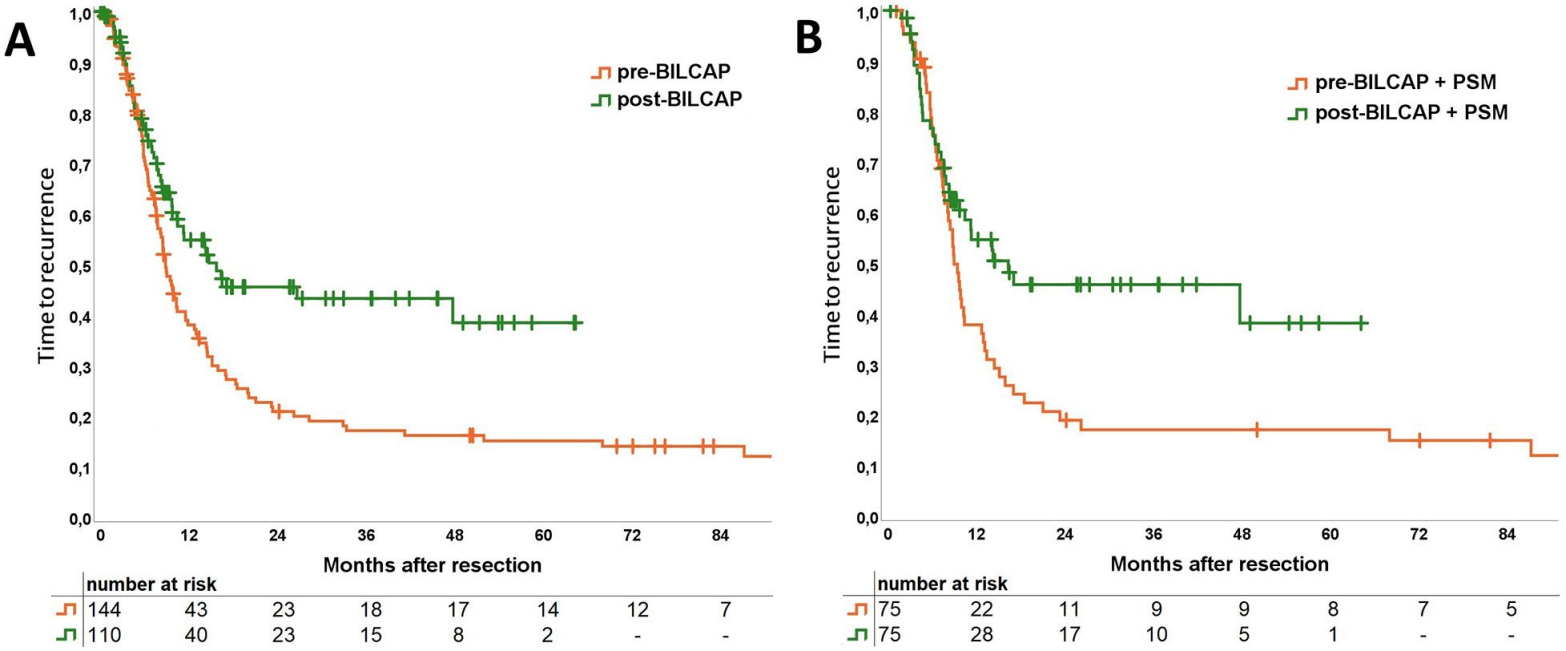
Kaplan Meyer curve comparing time to recurrence of **(A)** the initial pre- and post-BILCAP groups (*p* = 0.002) and **(B)** the pre- and post-BILCAP groups after propensity score matching (*p* = 0.019).

Regarding time to recurrence (TTR) the post-BILCAP group shows a significantly better TTR with a median TTR of 10.4 months versus 21.4 months for the pre-BILCAP group (*p* = 0.002, Figure 3A). After PSM the post-BILCAP group shows still a significantly longer TTR with a median of 21.3 months versus 10.8 months for the pre-BILCAP group (0.019, Figure 3B).

Median recurrence-free survival (RFS) was 8.8 months (CI95% 7.4–10.0) for the pre- and 11 months (CI95% 2.6–28.7) for the post-BILCAP group, showing a significant difference for the complete cohort (*p* = 0.005). Even after PSM median RFS was still significantly different with 8.6 (CI95% 7.5–10.3) and 10.7 (CI95% 0.00–42.1) months for the pre- and post-BILCAP group, respectively (*p* = 0.029).

## Discussion

The standard use of capecitabine as adjuvant therapy following the resection of cholangiocarcinoma remains a subject of active debate, and the findings of the BILCAP trial continue to be discussed controversially. At our center reflecting broader clinical practice in Germany, capecitabine has been the standard adjuvant treatment for patients undergoing curative-intended resection since 2018. This study aimed to provide “real-world” data independent of randomized controlled trials regarding the efficacy of capecitabine specifically for intrahepatic cholangiocarcinoma (ICC) within one of the largest Western single-center cohorts.

To account for the inhomogeneous distribution of confounding variables between the pre- and post-BILCAP eras, we utilized propensity score matching (PSM). Following this adjustment, confounders were homogeneous with no significant differences between groups. All standard mean differences of the matched parameters were <0.1, except N-status with −0.15. Balancing after PSM appears to be good. There were no significant differences in perioperative morbidity or mortality. Our analysis revealed that tumor recurrence was significantly lower in the post-BILCAP group. While overall survival (OS) did not show a statistically significant improvement after PSM, both time to recurrence (TTR) and recurrence-free survival (RFS) demonstrated significant long-term benefits in the post-BILCAP group, highlighting the potential value of adjuvant capecitabine.

Our cohort comprised 346 surgical explorations and 254 curative-intended resections. Most procedures were major (78%), with at least 39.4% qualifying as extended resections. Because ICC often presents at advanced stages, extensive surgery is frequently necessary rather than elective. The high volume of major resections in our study [over 40% when including vascular or visceral extensions, see data of our own group (19)] exceeds rates reported for example by Bergeat et al. (9) or a large multicenter cohort (20).

Postoperative morbidity was observed in 45.6% of cases, with major complications (Clavien-Dindo grade IIIa–IVb) occurring in 25.9%. The 90-day mortality rate was 8.7%. These figures are consistent with existing literature for ICC cohorts, which report morbidity frequencies ranging from 36% to 50.4% (7, 20). Exemplary Doussot et al. published data from a French multicenter analysis of initially 581 patients who underwent resections for ICC (21). Due to the aim of the study to analyze influence of morbidity on long-term survival perioperative deaths (*n* = 47, 8.1%) and palliative resections (*n* = 12, 2.1%) were excluded. Out of the remaining 522 patients, overall morbidity was 42.5% with a severe morbidity rate of 21.6%. The rate of major resection was 76.8% (*n* = 401) including 155 extended resections (29.7%) within the examination-group (21).

Furthermore, our cohort's 5-year OS of 22% sits at the lower end of the 21%–45% range reported in other studies (1, 2, 5, 22, 23). This discrepancy likely reflects the high proportion of advanced cases and extended resections in our population, whereas some higher-survival studies focused exclusively on R0 resections.

Regarding the impact of capecitabine on OS, the complete cohort showed a trend toward improved survival in the post-BILCAP era, though it did not reach statistical significance (*p* = 0.075). This closely mirrors the intention-to-treat results of the original BILCAP trial (13, 14). After PSM, the survival difference between groups further diminished (*p* = 0.291), suggesting that adjuvant capecitabine may not fundamentally extend OS in this setting.

However, exploratory analyses of the BILCAP data have suggested that N- and R-status significantly influence outcomes (14). In our subgroup analysis, while these factors did not significantly alter OS, they had a profound impact on TTR and RFS. Specifically, N0 and R0 patients in the post-BILCAP group derived the most significant benefit, suggesting that those with negative margins and no nodal involvement may be the best candidates for adjuvant capecitabine.

Tumor recurrence remains the primary barrier to long-term survival in ICC. Our overall recurrence rate of 60.6% aligns with literature benchmarks of 54.1%–70% (24–26). The significant improvement in TTR and RFS observed in our post-BILCAP group (*p* = 0.019 and *p* = 0.029, respectively) raises the question of why this does not translate into improved OS. One likely explanation is the shorter follow-up period for the post-BILCAP group (2018–2023) compared to the pre-BILCAP group (2008–2017).

Our findings contribute to a complex landscape of evidence. While the BILCAP trial reached significance only in its per-protocol analysis (13), other studies like PRODIGE-12 (comparing Gemcitabine/Oxaliplatin to surveillance) found no significant differences in OS or RFS (27). Large retrospective analyses have similarly failed to demonstrate a universal OS benefit for adjuvant chemotherapy, though benefits have been noted in high-risk subgroups such as those with T3/T4 stages or N1 status (28). As Qu et al. noted, the field remains “far from a clinical consensus” (29).

The treatment landscape for ICC is evolving rapidly with the introduction of immunotherapies and targeted agents. The TOPAZ-1 trial recently established the combination of Durvalumab, Gemcitabine, and Cisplatin as a standard for palliative care (30), and these approaches are increasingly being explored in neoadjuvant and conversion settings. Targeted therapies for FGFR2 fusions and IDH1/2 mutations are also becoming integral to management (31–33).

The single-center retrospective character of this study is one of its main limitations. PSM was applied to reduce this effect and raise validity. But potential residual confounding must be considered despite PSM. Comorbidities, performance status, systemic therapy, evolving patient selection and other parameters may vary. Further, the study was conducted as time-period comparison instead of a treatment-based analysis, which certainly may be subject to different bias. While surgical technique did not develop significantly over the time period, perioperative care, quality of imaging and staging as well as other systemic therapy options did evolve. This must be considered with uncertain influence. Further neoadjuvant therapy was applied in a small number of patients, distributed to PSM groups. The influence on survival is a further potential bias, and it was not adjusted explicitly for the propensity scores. Because of the time-period design the post-BILCAP group has a shorter follow-up. Within the pre-BILCAP group even some patients may have undergone adjuvant treatment, for example in the context of the early ACTICCA trial (Gemcitabine + Cisplatin versus observation) (34), which might be a bias. But the number of patients who received a recommendation for evaluation of inclusion is small (*n* = 8) and presumably half of these patients were drawn into the control arm. A few patients might have undergone adjuvant treatment due to recommendation of their own treating oncologist.

As described within the methods section we lack detailed information on adjuvant therapy data due to the reasons described. We cannot offer data regarding treatment exposure with capecitabine, dose, completion rates or any suffered toxicity. The analysis is based on the intention to treat, which is for sure a relevant bias. From our point of view, it nevertheless demonstrates the real-world reality.

In conclusion we found no significant OS benefit for adjuvant capecitabine on an intention-to-treat basis after ICC resection. However, the significant improvement in TTR and RFS suggests a clinical benefit that warrants further long-term observation.

## Data Availability

The raw data supporting the conclusions of this article will be made available by the authors, without undue reservation.
